# Formulation and *In Vitro* Evaluation of Bilayer Tablets of Nebivolol Hydrochloride and Nateglinide for the Treatment of Diabetes and Hypertension

**DOI:** 10.1155/2015/827859

**Published:** 2015-01-14

**Authors:** Harika Ryakala, S. Dineshmohan, Alluri Ramesh, V. R. M. Gupta

**Affiliations:** ^1^Vishnu Institute of Pharmaceutical Education and Research, Vishnupur, Narsapur, Medak, Telangana 502313, India; ^2^Department of Pharmaceutics, Pulla Reddy Institute of Pharmacy, Annaram, Hyderabad 502313, India

## Abstract

Diabetes mellitus (DM) and hypertension are two common diseases that often coexist. The most common cause of death in the diabetic patient is heart disease. In the present investigation we combine Nebivolol and Nateglinide for better patient compliance. IR layer was formulated using various superdisintegrants like Crospovidone, Croscarmellose sodium, and sodium starch glycolate and SR layer was formulated using polymers and gums like HPMC E_15_, ethyl cellulose, Gaur gum, and Xanthan gum. The disintegration and dissolution study of both layers showed that inclusion of surfactant (sodium lauryl sulphate) to the tablet formulation (IR) and dissolution medium (SR) enhanced the release of drugs from both layers. Kinetic studies of optimized IR layer (NBL8) and SR layer (N9) showed good linearity with regression coefficient of 0.9714 (Higuchi model) and 0.9931 (zero order kinetics), respectively. The above results reveal that the optimized IR layer of Nebivolol (NBL8) and SR layer of Nateglinide (N9) might be suitable for the treatment of diabetes and hypertension by sequential release of the two drugs in a bilayer tablet. IR-immediate release, SR-sustain release, NBL8-Nebivolol 8, N9-Nateglinide 9.

## 1. Introduction [1–11]

Type 2 diabetes mellitus and hypertension are among the most common chronic noncommunicable diseases and multifactorial disorders affecting both developed and developing countries. Lifestyle and genetic factors are responsible for diabetes mellitus and hypertension. In India, currently we have around 40 million cases of DM and it is projected to increase to 87 million by the year 2030. The prevalence of type 2 DM has raised from 1.2% to 11% over last three decades. A patient who suffers from type 2 DM has 2–4 times greater risk of death from cardiovascular causes than the patient without diabetes mellitus. The most common cause of death in the diabetic patient is heart disease. In addition, peripheral vascular disease, end-stage renal disease, blindness, and amputations are common comorbidities in diabetic patients. Therefore, combination therapy is important for the prevention of diabetes in patients with hypertension. Nebivolol hydrochloride is a BCS class-2 drug and *β*_1_ receptor blocker with nitric oxide-potentiating vasodilator effect. It is used in treatment of hypertension and is highly cardioselective. It lowers blood pressure by reducing peripheral vascular resistance and significantly increases stroke volume with preservation of cardiac output. Nebivolol has reduced typical beta-blocker related side effects such as fatigue, clinical depression, bradycardia, and impotence. Nateglinide is a novel D-phenylalanine derivative that inhibits ATP-sensitive K+ channels in pancreatic beta-cells in the presence of glucose and thereby stimulates the prandial release of insulin. It is selective blocker of pancreatic beta-cells with a short half-life of 1.5–2.5 hrs. Therefore, in order to prolong its effect in the body and to decrease oscillations in concentration level in plasma, a modified drug delivery system is needed for Nateglinide.

The aim of present research work was undertaken to formulate bilayer tablets of Nebivolol and Nateglinide through its incorporation of an oral dosage form that is able to release Nebivolol immediately as well as sustained release of Nateglinide for 12 hrs to enhance the oral bioavailability of Nateglinide. The main objective of this work was formulation of bilayer tablets composed of two different classes of drugs by using a simple and easy-to-scale-up formulation strategy.

## 2. Materials and Methods

Nateglinide and Nebivolol were obtained as gift samples from Hetero Drugs Ltd. Hyderabad, India. Xanthan gum, Guar gum, and HPMC E15 were gifted by Ashland Labs, Hyderabad, India. Sodium starch glycolate, Crospovidone, and Croscarmellose sodium were procured from SD Fine Chemicals, Mumbai, India. Talc and magnesium stearate were purchased from Nice Chemie Pvt. Ltd., Mumbai, India. Ethyl cellulose N 50 was gifted by Aurobindo Pharma, Hyderabad, India.

### 2.1. Characterization of IR/SR Granules [12–14]

Solubility studies of the drugs were carried out in various aqueous solutions and buffers. Drug excipient compatibility studies were done using FTIR. The granules of both the layers of IR/SR were evaluated for various precompression parameters. The angle of repose was measured by fixed funnel method. Bulk and tapped densities were determined by tapped density apparatus from which compressibility index and Hausner's ratio values were calculated and from the values obtained flow property of granules was determined from Table 1.

#### 2.1.1. Drug-Excipient Compatibility Studies by FT-IR

The compatibility of drugs with their respective excipients was studied by FT-IR spectroscopy (Model number 02437, Shimadzu, India). The scanning was performed 20 times at scanning speed 2 mm/sec with resolution of 4 cm^−1^ over the region 4000–400 cm^−1^. The scans were evaluated for presence of principle peaks of drug, shifting and masking of drug peaks, and appearance of new peaks due to polymer interaction.

### 2.2. Analytical Method Development [15–18]

#### 2.2.1. Construction of Calibration Curve of Nebivolol and Nateglinide

Standard dilutions were prepared in the range of 2–10 *μ*g/mL using 0.1 N HCl for Nebivolol and absorbance was determined at *λ*_max⁡_ (282 nm) in UV spectrophotometer (UV-1700, Shimadzu, India). Similarly standard dilutions were prepared in the range of 2–10 *μ*g/mL using 0.01 N HCl with 0.5% w/v SLS for Nateglinide and absorbance was determined at *λ*_max⁡_ (210 nm) in UV spectrophotometer. From the values obtained, standard graph can be plotted between concentration and absorbance values.

SLS: Sodium Lauryl Sulfate.

### 2.3. Preparation of Immediate Release Nebivolol Tablets [19]

Immediate release layer of Nebivolol (NBL1–NBL9) was prepared by direct compression method. Nebivolol and other excipients like microcrystalline cellulose, Crospovidone, Croscarmellose sodium, and sodium starch glycolate and sodium lauryl sulfate were accurately weighed and sifted through sieve #40 and mixed in a polybag and these formulations are given in Table 2. The sifted powders were thoroughly mixed for approximately 5 min and again passed through sieve #40 to get uniform particle size. Magnesium stearate was added into the powder mixture for lubrication after passing through sieve #40 and 0.125% w/w of iron oxide red previously sifted to sieve #100 was added to the above mixture and blended thoroughly to ensure uniform color.

### 2.4. Preparation of Sustained Release Nateglinide Tablets [19]

Sustained release layer of Nateglinide (N1–N13) was prepared by wet granulation technique by adding 5% concentration of PVP K 30 dissolved in isopropyl alcohol as a binding agent and these formulations are represented in the Table 3. Required quantities of Nateglinide and other excipients like HPMC E_15_, Guar gum, Xanthan gum, ethyl cellulose, mannitol, and microcrystalline cellulose were weighed accurately and were sifted through sieve #40 and were mixed thoroughly and a sufficient volume of binding agent was added slowly to get cohesive mass. Then mass was passed through sieve #20 to obtain the granules. Next the granules were dried at 50°C in a hot air oven until dry the dried granules were lubricated uniformly with magnesium stearate; then talc was added and mixed properly. The above granules were compressed into tablets by 10-station tablet compression machine (Mini Press I, Karnavati, Gujarat, India) using 9 mm punch. In batches N1 to N3, HPMC E_15_ was used as the sustained release polymer and in batches N4 to N7 Guar gum was used, and in N8 Xanthan gum, in N9 combination of HPMC E_15_ and Guar gum, and in N10 to N12 combination of Guar gum and ethyl cellulose, and finally in N13 combination of Guar gum and Xanthan gum was used as sustained release polymer.

### 2.5. Preparation of Bilayer Tablets [19]

In order to prepare bilayer tablets, the dissolution test was conducted for both layers of IR/SR separately with the aim of selecting the best formulations. Based on dissolution behavior, formulations NBL-8 and N-9 were selected for bilayer tablet. First, sustained release Nateglinide layer was placed in the die cavity and punched with low compression force. Then the immediate release Nebivolol layer was placed in the die cavity and allowed for punching with optimum hardness of 6–8 kg/cm^2^ to form bilayer tablets. Compression was made by using 10 mm punches (Mini Press I, Karnavati, Gujarat, India). The total weight of each bilayer tablet was adjusted to 450 mg, containing 10 mg of Nebivolol in fast-release layer and 60 mg of Nateglinide in sustained release layer. Prepared bilayer tablets were evaluated for various postcompression parameters and* in vitro* dissolution studies.

### 2.6. Evaluation of IR/SR Tablets [19–22]

The prepared tablets were subjected to various evaluation tests like thickness, hardness, weight variation, friability, and drug content. Thickness of the tablets was determined by using vernier calipers. Randomly 10 tablets were selected and used for determination of thickness. Hardness is termed as the tablet crushing strength and it is the force required to break a tablet diametrically. Hardness of tablets was measured by selecting 6 tablets randomly and the hardness of each tablet was measured with Monsanto hardness tester. The hardness was noted. The hardness is usually measured in terms of kg/cm^2^. For weight variation test individual weight of 20 tablets was taken; then their average weight and their mean and standard deviation were calculated and compared with the standards. The weight of the tablet being made is measured to ensure that it contains predetermined amount of drug. The tablet friability is a measure of loss due to abrasion. The preweighed tablets were exposed to repeated shocks in Roche friabilator in which they are initially weighed (*W*_0_) and kept in a tumbling and rotating apparatus drum and were subjected to fall from 6 inches height. After completion of 100 rotations, the tablets were reweighed (*W*) and the percent loss in weight or friability (*f*) was calculated by the formula given below:

(1) $$\begin{array}{c} \%\text{Friability}=\frac{\text{Initial}\;\text{weight}-\text{Final}\;\text{weight}}{\text{Initial}\;\text{weight}}\times 100. \end{array}$$

### 2.7. Drug Content [23]

Twenty tablets were selected randomly and average weight was calculated. The tablets were crushed in a mortar and accurately weighed amount of average tablet weight was taken from the crushed blend and transferred in to a 100 mL volumetric flask. To this little amount of methanol was added to dissolve the drug and volume was made up to the mark with concerned medium. The content was shaken periodically and kept for 1 hour to allow the drug to dissolve completely. Then it was filtered and appropriate dilutions were made. Finally dilutions were observed using spectrophotometer to determine % drug content. The drug content should be within the range between 90 and 110% of standard amount.

### 2.8. Disintegrating Time [24]

The disintegration test is carried out in an apparatus (Electro lab, Mumbai) containing a basket rack assembly with six glass tubes of 7.75 cm in length and 2.15 mm in diameter, the bottom of which consists of a #10 mesh sieve. The basket is raised and lowered 28–32 times per minute in a medium of 900 mL water which is maintained at 37 ± 2°C. Six tablets were placed in each of the tubes and the time required for complete passage of tablet fragments through the mesh (#10) was considered as the disintegration time of the tablet.

### 2.9. *In Vitro* Dissolution Studies [17, 18]

The release of drug from different batches of prepared tablets was studied using USP dissolution apparatus type II. The dissolution medium used was 500 mL of 0.1 N HCl for first 30 minutes for immediate release layer and then 900 mL of 0.01 N HCl with 0.5% w/v SLS was used up to 12 hours for sustained release layer. The temperature was maintained at 37 ± 0.5°C and the stirring rate was 50 rpm. The samples were withdrawn at regular intervals and this withdrawn volume was replaced with fresh medium. The collected samples were filtered using Whatman filter paper and observed using spectrophotometer at respective *λ*_max⁡_ against a blank (respective medium).

### 2.10. Evaluation of Bilayer Tablet [17, 18, 25]

Evaluation parameters of bilayer tablet were performed according to I.P. specifications. Parameters such as weight variation were performed by taking average weight of 20 tablets and hardness test was performed by Monsanto hardness tester. Thickness of the tablet was measured using vernier caliper. Friability test was performed by taking 6 tablets in Roche friabilator and % friability was calculated.* In vitro* drug release studies of bilayer tablets were carried out uvusing USP dissolution apparatus type II in 500 mL of 0.1 N HCl for first 30 minutes and in 900 mL of 0.01 N HCl with 0.5% SLS up to 12 hours. Samples were collected at regular intervals of time and filtered. The medium in bowl was discarded after 30 minutes and replaced with another medium which was preferred for dissolution of sustain release layer. The collected samples were filtered and observed in UV spectrophotometer. The results were calculated using simultaneous equation method as given below:

(2) Cx=A2ay1−A1ay2ax2ay1−ax1ay2,Cy=A1ax1−A2ax2ax2ay1−ax1ay2,

where

 
*Cx* and *Cy* are concentrations of Nebivolol and Nateglinide,
 
*A*_1_ is absorbance value at wavelength *λ*_1_,
 
*A*_2_ is absorbance value at wavelength *λ*_2_,
 
*ax*_1_ is absorptive value of Nebivolol at *λ*_1_,
 
*ax*_2_ is absorptive value of Nebivolol at *λ*_2_,
 
*ay*_1_ is absorptive value of Nateglinide at *λ*_1_,
 
*ay*_2_ is absorptive value of Nateglinide at *λ*_2_.

### 2.11. Kinetic Data Analysis [15, 19]

The drug release kinetic studies were carried out for bilayer tablets of Nebivolol and Nateglinide and were evaluated using the linear regression method:

1. zero order kinetic model—cumulative % of drug released versus *T*;
2. first order kinetic model—log cumulative percent drug remaining versus *T*;
3. Higuchi's model—cumulative percent drug released versus square root of *T*;
4. Korsmeyer equation/Peppa's model—log cumulative percent drug released versus log *T*.

### 2.12. Stability Studies [19]

The purpose of stability testing is to provide evidence on how the quality of drug product varies with time under the influence of a variety of environmental factors such as temperature, humidity, and light. The optimized bilayer tablets were subjected to stability studies (as per ICH guidelines) at 40°C ± 2°C/75% ± 5% RH in a humidity chamber. The products were evaluated for their physical characteristics and* in vitro* drug release profiles over a period of 3 months.

## 3. Results and Discussion

### 3.1. Analytical Method Development

Calibration curves were plotted for both Nebivolol and Nateglinide based on the data obtained in UV spectrophotometer and these curves showed regression coefficient of 0.9999 and 0.9996 for the respective drugs.

#### 3.1.1. Construction of Calibration Curve of Nebivolol and Nateglinide

**Figure 1:**
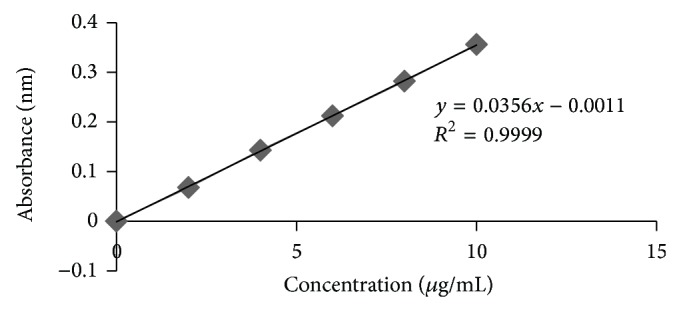
Calibration curve of Nebivolol in 0.1 N HCl.

**Figure 2:**
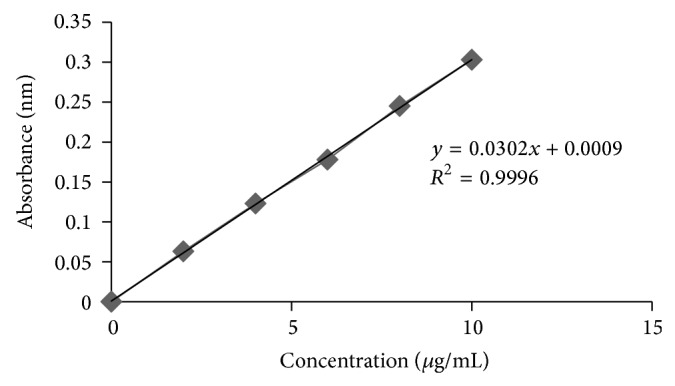
Calibration curve of Nateglinide in 0.01 N HCl with 0.5% w/v SLS.

See Tables 5 and 6 and Figures 1 and 2.

### 3.2. Characterization of IR/SR Granules

#### 3.2.1. Solubility Studies

Solubility studies were carried out for both Nebivolol and Nateglinide in various solvents and buffers and results were found to be as in Table 7.

#### 3.2.2. Drug-Excipient Compatibility Studies by FT-IR

**Figure 3:**
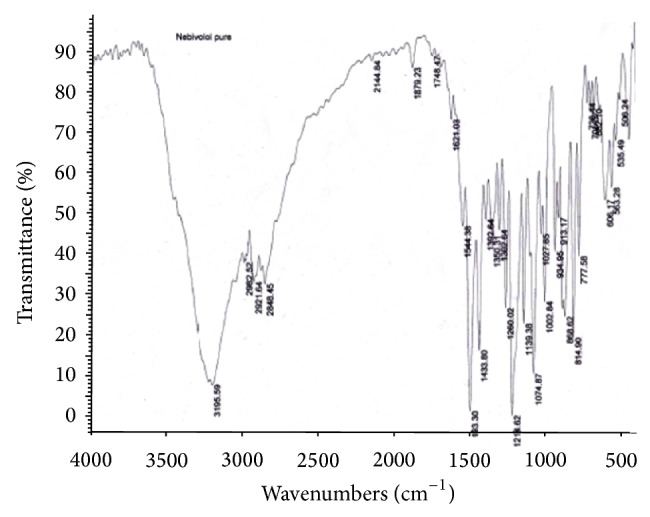
FT-IR (Nebivolol pure drug).

**Figure 4:**
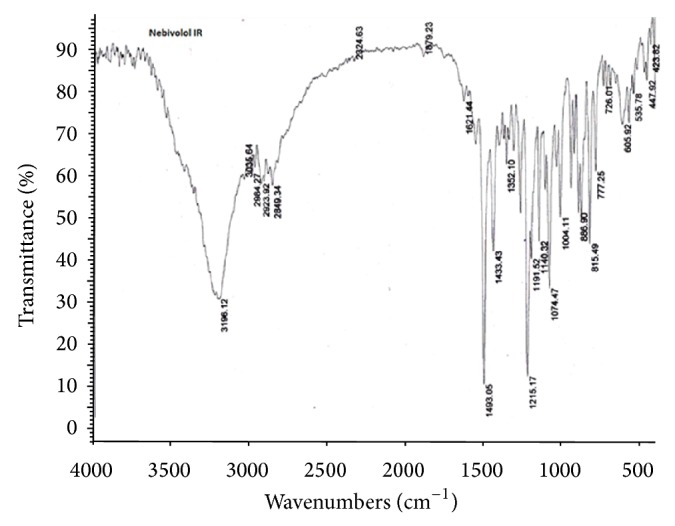
FT-IR (Nebivolol optimized formula).

**Figure 5:**
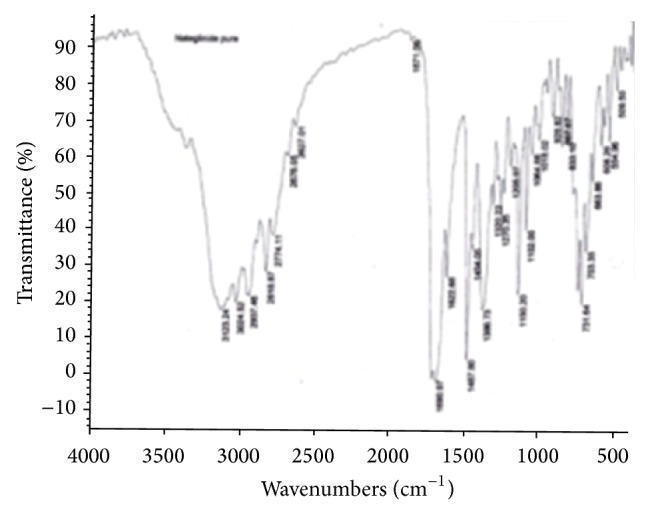
FT-IR (Nateglinide pure drug).

**Figure 6:**
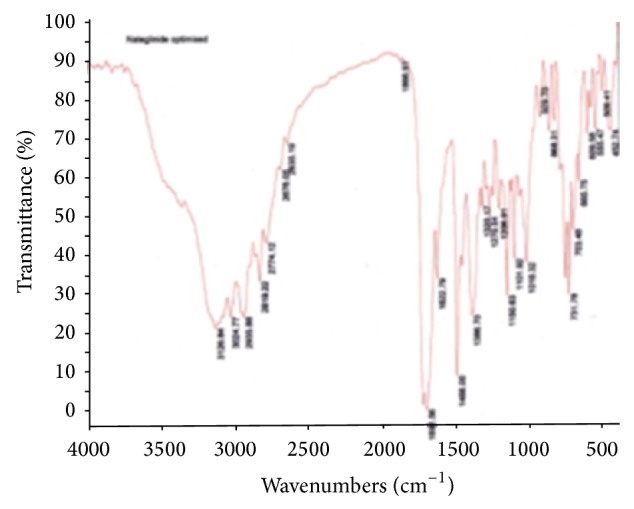
FT-IR (Nateglinide optimized formula).

As part of compatibility studies, FT-IR studies were performed as shown in Figures 3, 4, 5, and 6 for both drugs along with the excipients to detect any major interference between drug and excipients.

As per the data tabulated in Tables 8 and 9, there was no significant shift in the positions of wave numbers when compared to that of the pure drugs. As there is no interaction observed between the drugs and excipients of the formulations, these excipients were chosen for the formulations.

#### 3.2.3. Micromeritics Studies

Flow properties of Nebivolol and Nateglinide were carried out and results are shown in the Tables 10 and 11 which were found to be as per the limits given in the Table 1.

### 3.3. Evaluation of IR/SR Tablets

**Figure 7:**
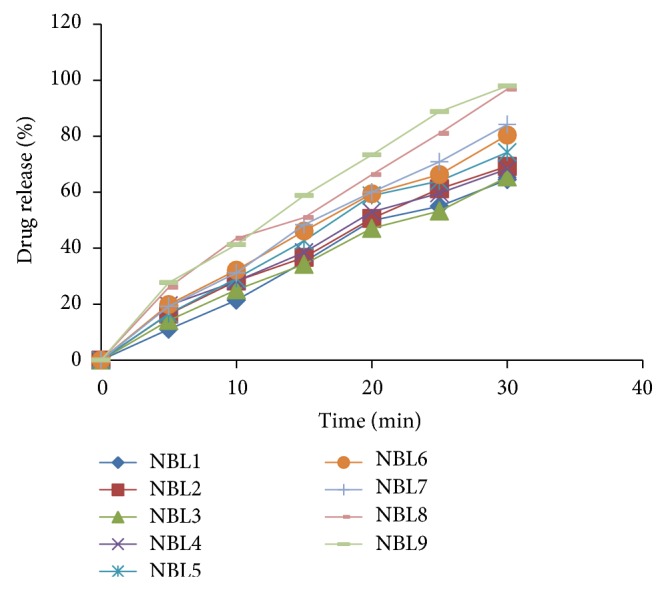
Comparison of % drug release of Nebivolol immediate release tablets (NBL1–NBL9).

Immediate release tablets of Nebivolol were successfully prepared by direct compression method using various concentrations of superdisintegrants like Crospovidone, Croscarmellose sodium, sodium starch glycolate, and other tableting excipients. The prepared formulations were evaluated for postcompression parameters. The results of all formulations were found to be within limits (weight variation ±7.5%, hardness range 4.5–7 kg/cm^2^, friability <1%, drug content 90–110%, and disintegration time 5–30 minutes) and all the values were reported in Table 12.* In vitro* drug release studies of immediate release tablets were carried out using USP type II dissolution apparatus in 500 mL of 0.1 N HCl at 50 rpm up to 30 minutes. Except formulation code NBL7, all other formulations contain same concentrations of different superdisintegrants (5–10%). Formulation NBL7 has increased amount (12.5%) of sodium starch glycolate as a superdisintegrant. Formulation batches NBL1–NBL7 have shown an immediate release profile between 64 and 84% within 30 minutes. The formulations NBL1–NBL6 showed lesser drug release 64–80% and NBL7 showed 84% of drug release. Hence to further increase the drug release SLS was added in NBL8 and NBL9 and both formulations had shown good drug release 96-97% at the end of 30 minutes. From that above data formulation NBL8 was optimized for bilayer tablet as it reflects good disintegration and dissolution characters with lesser amount of superdisintegrant (5%). These results are represented in Table 13 and Figure 7.

**Figure 8:**
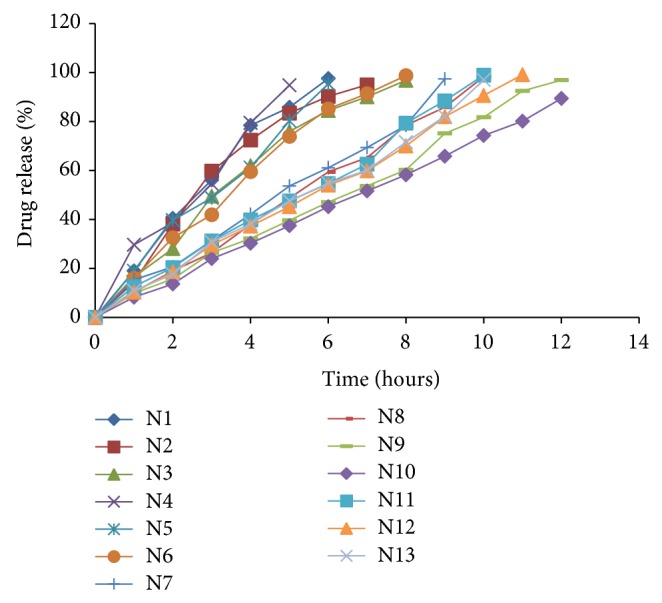
Comparison of % drug release of Nateglinide sustained release tablets (N1–N13).

Nateglinide sustained release layer of bilayer tablet was prepared by wet granulation method using common granulating agent and diluent for all the formulations, that is, 5% PVP +IPA and MCC, respectively, having varying concentrations of polymers and gums which were used either individually or in combination, with N13 as an exception in which combination of diluents such as Mannitol and MCC was used. Postcompression parameters were evaluated for all the formulations and results were found to be within the limits as mentioned in above section and were shown in Table 14.* In vitro* drug release studies were carried out in 900 mL of 0.01 N HCl with 0.5% w/v SLS for 12 hours at 50 rpm and 37 ± 0.5°C using USP dissolution apparatus type II. The formulations N1–N8 in which either polymer or gum was used individually in the range of 10–25% had shown drug release in the range of 10–98% in 10 hours. In the above formulations as the single retarding agent was used and even its amount was less, release was not controlled up to 12 hours; hence formulations N9–N13 were prepared with different combinations of polymers and gums. In the formulations N11 and N12 containing same amount of guar gum (20%) and increasing amount of ethyl cellulose, respectively (5% and 10%), drug release was not sustained up to 12 hours because of less amount of ethyl cellulose. Similar effect was observed for N13 which contain same material as N11 with an exception in diluent where combination of diluents MCC and Mannitol was used. Here it implies that the change in diluent has no effect on drug release. For the formulation N10 containing two natural gums, that is, Guar gum (20%) and Xanthan gum (15%) in combination, drug release was found to be less in 12 hours when compared to the formulation N9 in which HPMC (20%) and Xanthan gum (15%) were used in combination. Here it can be understood that more retardation of drug release was observed when two natural gums were used in combination (N10) compared to combination of a polymer and natural gum (N11, N12, N13, and N9). But here N9 formulation was optimized as it achieves the objective by showing maximum drug release (97%) in 12 hours and all the results are represented in Table 15 and Figure 8.

### 3.4. Evaluation of Bilayer Tablets

**Figure 9:**
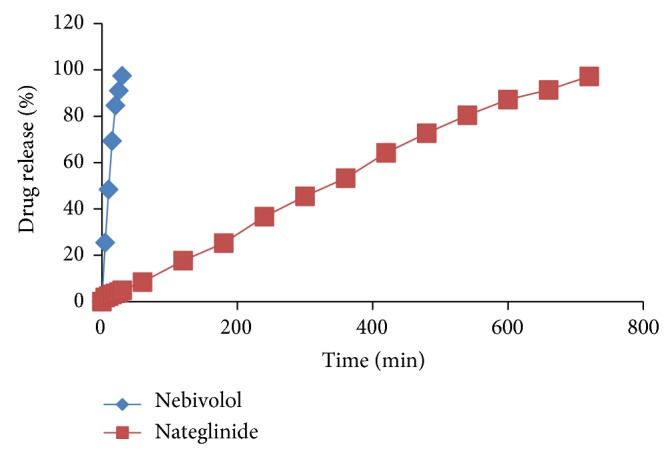
Cumulative % drug release of Nebivolol and Nateglinide bilayer tablet.

Bilayer tablets were prepared successfully after selecting the optimized formulations of immediate release layer (NBL8) and sustain release layer (N9) using 10 mm punches (Mini Press I, Karnavati, Gujarat, India). The prepared bilayer tablets were evaluated for postcompression parameters and results were found to be within the limits mentioned in the above section and were shown in Table 16.* In vitro* drug release studies of bilayer tablets were carried out using USP dissolution apparatus type II in 500 mL of 0.1 N HCl for first 30 minutes and in 900 mL of 0.01 N HCl with 0.5% SLS up to 12 hours. From the results, drug release of Nebivolol immediate release layer was found to be 97.43% in 30 minutes and that of the Nateglinide sustain release layer was 97.22% at the end of 12 hours and % drug release of Nateglinide in first half hour was found to be 4.9%. It implies that the release of sustain release drug in the medium preferred for immediate release layer was found to be negligible and thus shows no irregularities in the drug release of bilayer tablet and values are represented in the Table 17 and Figure 9.

### 3.5. Kinetic Data Analysis

**Figure 10:**
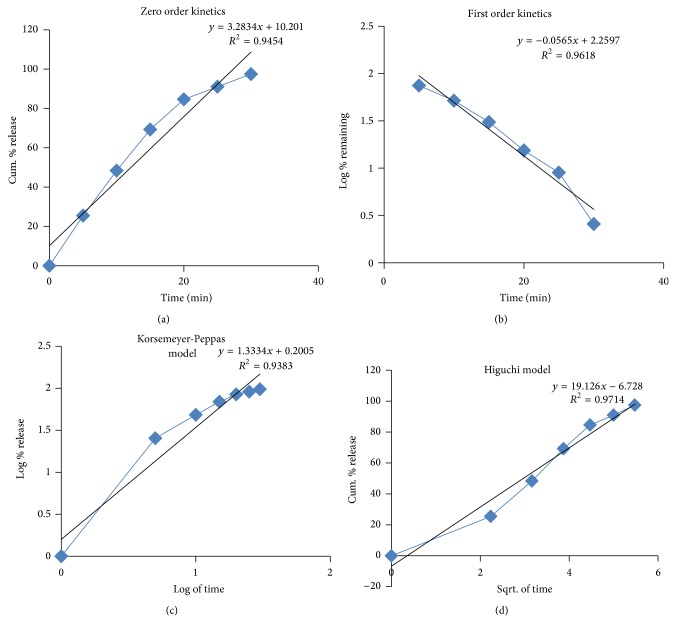
Drug release kinetics of Nebivolol (immediate release) layer in bilayer tablet.

The drug release kinetic data of Nebivolol layer is shown in Table 18 and graphs are represented in Figure 10. From the graphical representation it can be understood that this layer is best fit in to Higuchi model which had shown a regression coefficient (*R* ^2^) of 0.9714. The results of the* in vitro* release data of this layer were fitted to the Korsemeyer-Peppas equation given in Table 4 to analyze the release pattern of the drug from the polymeric system. The value of “*n*” was found to be more than 0.89, indicating the drug release follows super case-II transport.

**Figure 11:**
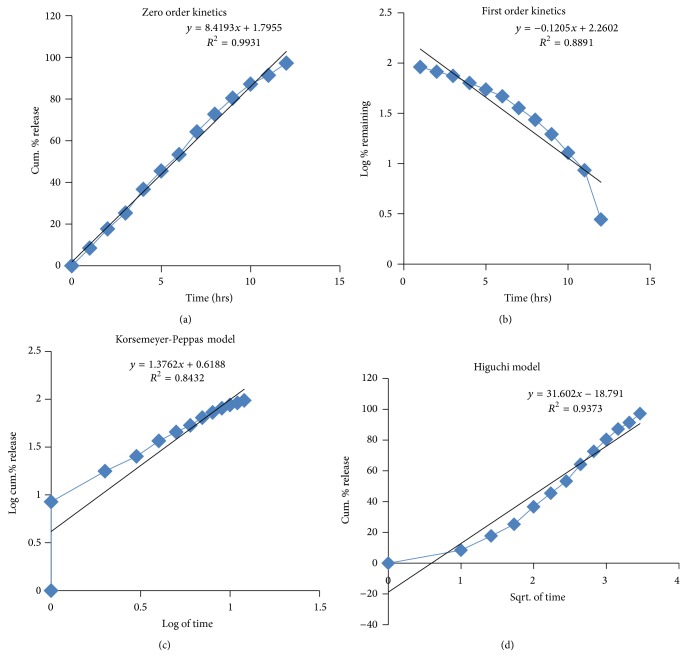
Drug release kinetics of Nateglinide (sustain release) layer in bilayer tablet.

The drug release kinetic data of Nateglinide layer is shown in Table 19 and graphs are represented in Figure 11. From the graphical representation it can be understood that this layer is best fit in to zero order kinetics which had shown a regression coefficient (*R* ^2^) of 0.9931. The results of the* in vitro *release data of this layer were fitted to the Korsemeyer-Peppas equation to analyze the release pattern of the drug from the polymeric system. The value of “*n*” was found to be more than 0.89, indicating the drug release follows super case-II transport.

### 3.6. Stability Studies

#### 3.6.1. Condition: 40°C/75% RH

See Table 20.

#### 3.6.2. Test Frequency: 0 Months, 1 Month, 2 Months, and 3 Months

- All physical and chemical parameters were found to be satisfactory based on the stability data.
- Photostability studies have shown that the medicinal product is non-light sensitive.

## 4. Conclusion 

The present study demonstrated the successful formulation and evaluation of an antidiabetic and antihypertensive in a single dosage form as bilayer tablet. In the bilayer tablet, immediate release layer of Nebivolol was prepared by direct compression method using various super disintegrants in which optimized formula (NBL8) contains sodium starch glycolate as super disintegrant and sustain release layer of Nateglinide was prepared by wet granulation method using different release retarding agents in which optimized formula (N8) contains combination of HPMC and Xanthan gum as release retardants. The drug excipient compatibility studies carried out using FTIR revealed that there was no interaction found between drugs and excipients. All the pre- and postcompression studies revealed that the results were found to be within the official limits.* In vitro* release studies reveal that Nebivolol immediate release layer in bilayer tablet was found to be 97.43% within 30 minutes and Nateglinide sustained release layer was 97.22% at the end of 12 hrs. Release kinetics showed good linearity by best fitting in to Higuchi model for IR layer and zero order kinetics for SR layer and stability studies showed no changes after exposing to accelerated conditions for a period of 3 months with respect to physical characteristics and* in vitro* drug release studies.

From the above study, it can be concluded that the prepared bilayer tablets achieve the objective of the research work in treating the diabetes and hypertension with the sequential release of two drugs. As these tablets are supposed to be given twice a day which reduces the dosage frequency and are cost effective, it can be best alternative to conventional dosage forms having more frequency of administration.

## Figures and Tables

**Table 1:** Scale of flowability (USP).

Flow character	Angle of repose (*θ*)	Compressibility index (%)	Hausner's ratio
Excellent	25–30	≤10	1.00–1.11
Good	31–35	11–15	1.12–1.18
Fair	36–40	16–20	1.19–1.25
Passable	41–45	21–25	1.26–1.34
Poor	46–55	26–31	1.35–1.45
Very poor	56–65	32–37	1.46–1.59
Very, very poor	>66	>38	>1.60

**Table 2:** Composition of Nebivolol immediate release tablets (150 mg).

Batch code	Ingredients in mg/tablet							
	Drug	MCC	CP	CCS	SSG	Mg. stearate	SLS	Fe_2_O_3_
NBL-1	10	131	7.5	—	—	1.5	—	Qs
NBL-2	10	123.5	15	—	—	1.5	—	Qs
NBL-3	10	131	—	7.5	—	1.5	—	Qs
NBL-4	10	123.5	—	15	—	1.5	—	Qs
NBL-5	10	131	—	—	7.5	1.5	—	Qs
NBL-6	10	123.5	—	—	15	1.5	—	Qs
NBL-7	10	119.5	—	—	18.75	1.5	—	Qs
NBL-8	10	131	—	—	7.5	1.5	0.3	Qs
NBL-9	10	123.5	—	—	15	1.5	0.3	Qs

MCC: microcrystalline cellulose, CP: Crospovidone, CCS: Croscarmellose sodium, SSG: sodium starch glycolate, SLS: sodium lauryl sulfate.

**Table 3:** Composition of Nateglinide sustained release tablets.

Ingredients	N1	N2	N3	N4	N5	N6	N7	N8	N9	N10	N11	N12	N13
Drug	60	60	60	60	60	60	60	60	60	60	60	60	60
HPMC	30	45	60	—	—	—	—	—	45	—	—	—	—
Guar gum	—	—	—	30	45	60	75	—	—	60	60	60	60
X-gum	—	—	—	—	—	—	—	75	45	45	—	—	—
EC	—	—	—	—	—	—	—	—	—	—	15	30	15
Mannitol	—	—	—	—	—	—	—	—	—	—	—	—	72
MCC	180	165	150	180	165	150	135	135	120	129	135	120	72
PVP	15	15	15	15	15	15	15	15	15	15	15	15	15
Mg. st.	7.5	7.5	7.5	7.5	7.5	7.5	7.5	7.5	7.5	6	7.5	7.5	6
Talc	7.5	7.5	7.5	7.5	7.5	7.5	7.5	7.5	7.5	—	7.5	7.5	—
Total wt.	**300**	**300**	**300**	**300**	**300**	**300**	**300**	**300**	**300**	**300**	**300**	**300**	**300**

HPMC: hydroxy propyl methyl cellulose; X-gum: Xanthan gum; EC: ethyl cellulose; MCC: microcrystalline cellulose; PVP: polyvinyl pyrrolidone, Mg. st.: magnesium stearate.

**Table 4:** Diffusion exponent and solute release mechanism (Korsemeyer equation/Peppas model).

Diffusion exponent (*n*)	Drug release mechanism
0.45	Fickian diffusion
0.45 < *n* = 0.89	Anomalous (non-Fickian) diffusion
0.89	Case-II transport
*n* > 0.89	Super case-II transport

**Table 5:** Data for calibration curve of Nebivolol in 0.1 N HCl.

Concentration (*μ*g/mL)	Absorbance (nm)
0	0
2	0.068
4	0.143
6	0.212
8	0.282
10	0.356

**Table 6:** Data for calibration curve of Nateglinide in 0.01 N HCl with 0.5% w/v SLS.

Concentration (*µ*g/mL)	Absorbance (nm)
0	0
2	0.063
4	0.123
6	0.178
8	0.245
10	0.303

**Table 7:** Solubility study data of Nebivolol and Nateglinide in various solvents and buffers.

Name of solvent/buffer	Nebivolol (mg/mL)	Nateglinide (mg/mL)
Water	0.0017	0.001
HCl buffer pH 1.2	1.2234	0.105
Phosphate buffer pH 6.8	0.0012	0.021
PEG	0.9102	—
DMSO	0.4567	0.574
Methanol	0.0814	0.342

**Table 8:** Characteristic peak of Nebivolol pure drug and optimized formula.

Functional groups	Reported value (cm^−1^)	Observed value (cm^−1^) Pure drug	Observed value (cm^−1^) Optimized formula
COOH	3300–2500	3195.59	3196.12
Nitro compounds	1550–1475	1493.30	1493.05
Alkyl halides	1300–1150	1214.62	1215.17
Aliphatic amines	1250–1020	1074.87	1074.47

**Table 9:** Characteristic peak of Nateglinide pure drug and optimized formula.

Functional groups	Reported value (cm^−1^)	Observed value (cm^−1^) Pure drug	Observed value (cm^−1^) Optimized formula
COOH	3300–2500	3123.24	3126.84
Carbonyls	1760–1665	1690.97	1691.56
Nitro compounds	1550–1475	1487.80	1488.00
Aliphatic amines	1550–1475	1150.20	1150.63

**Table 10:** Flow properties of Nebivolol immediate release layer.

Batch code	Angle of repose	Bulk density (g/cm^3^)	Tapped density (g/cm^3^)	Carr's index (%)	Hausner's ratio
NBL-1	30.17	0.34	0.40	15.00	1.17
NBL-2	34.70	0.35	0.47	25.53	1.34
NBL-3	26.10	0.34	0.41	17.07	1.20
NBL-4	29.12	0.34	0.40	15.00	1.17
NBL-5	28.47	0.34	0.39	12.82	1.14
NBL-6	26.96	0.35	0.43	18.60	1.22
NBL-7	26.10	0.36	0.41	12.19	1.13
NBL-8	**27.92**	**0.35**	**0.42**	**16.66**	**1.20**
NBL-9	28.01	0.35	0.40	12.50	1.14

**Table 11:** Flow properties of Nateglinide sustained release layer.

Batch code	Angle of repose	Bulk density (gm/cm^3^)	Tapped density (gm/cm^3^)	Carr's index (%)	Hausner's ratio
N1	35.12	0.32	0.37	13.50	1.15
N2	32.15	0.31	0.40	22.50	1.29
N3	31.18	0.34	0.38	10.52	1.11
N4	32.20	0.38	0.44	13.63	1.15
N5	34.80	0.30	0.35	14.28	1.16
N6	33.15	0.36	0.42	14.28	1.16
N7	30.00	0.29	0.33	12.12	1.13
N8	28.55	0.30	0.35	14.28	1.16
N9	**27.50**	**0.31**	**0.35**	**11.42**	**1.13**
N10	31.85	0.39	0.46	15.21	1.18
N11	27.90	0.34	0.40	15.00	1.17
N12	34.55	0.39	0.45	13.33	1.15
N13	34.41	0.33	0.38	13.15	1.15

**Table 12:** Evaluation parameters of Nebivolol immediate release tablets.

Batch code	Weight variation (%)	Hardness (kg/cm^2^)	Thickness (mm)	Friability (%)	Drug content (%)	Disintegration time (min)
NBL-1	0.53	4.3	2.83	0.13	95.56	3.40
NBL-2	1.19	4.5	3.29	0.15	97.20	2.55
NBL-3	2.51	4.0	3.18	0.14	96.45	4.10
NBL-4	1.19	5.0	3.30	0.13	90.84	3.28
NBL-5	1.25	3.5	2.85	0.16	103.01	3.56
NBL-6	3.18	4.3	2.91	0.12	97.62	2.45
NBL-7	0.53	4.2	3.10	0.15	100.94	1.50
NBL-8	**1.25**	**4.5**	**3.05**	**0.18**	**98.19**	**3.45**
NBL-9	0.53	4.5	3.20	0.16	99.42	2.40

**Table 13:** *In vitro* drug release data of Nebivolol immediate release tablets (NBL1–NBL9).

Time	NBL-1	NBL-2	NBL-3	NBL-4	NBL-5	NBL-6	NBL-7	NBL-8	NBL-9
0	0	0	0	0	0	0	0	**0**	0
5	11 ± 3.12	16 ± 3.26	14 ± 3.11	19 ± 3.80	16 ± 3.57	19 ± 3.85	19 ± 24	**25 ± 3.96**	27 ± 3.68
10	21 ± 3.45	28 ± 3.40	25 ± 4.25	28 ± 3.54	29 ± 4.20	32 ± 3.15	31 ± 3.20	**43 ± 3.56**	41 ± 3.24
15	35 ± 3.08	36 ± 4.05	34 ± 4.50	38 ± 3.69	42 ± 4.22	46 ± 4.12	48 ± 4.01	**50 ± 3.94**	58 ± 3.80
20	49 ± 4.15	50 ± 4.91	47 ± 3.75	52 ± 4.52	58 ± 3.75	59 ± 3.46	60 ± 4.23	**66 ± 4.25**	73 ± 4.31
25	55 ± 3.89	61 ± 3.89	53 ± 3.90	59 ± 3.96	64 ± 3.58	66 ± 4.29	70 ± 3.99	**80 ± 3.98**	88 ± 3.78
30	64 ± 4.52	69 ± 3.68	65 ± 4.82	68 ± 4.95	74 ± 4.52	80 ± 4.42	84 ± 4.58	**96 ± 3.80**	97 ± 3.94

**Table 14:** Evaluation parameters of Nateglinide sustained release tablets.

Batch code	Weight variation (%)	Hardness (kg/cm^2^)	Thickness (mm)	Friability (%)	Drug content (%)
N1	0.22	7.0	3.97	0.59	96.12
N2	0.89	5.8	3.95	0.62	97.45
N3	0.55	7.2	3.99	0.60	98.10
N4	1.55	6.8	3.85	0.54	95.53
N5	1.20	6.2	3.57	0.70	98.63
N6	0.89	5.5	3.84	0.69	98.84
N7	0.22	6.5	3.73	0.72	97.14
N8	0.22	5.5	3.69	0.54	101.15
N9	**0.55**	**6.8**	**3.89**	**0.52**	**98.68**
N10	1.55	5.4	3.91	0.63	102.42
N11	0.89	6.5	4.11	0.71	97.85
N12	1.88	7.0	3.90	0.51	96.81
N13	1.20	6.0	3.66	0.56	100.65

**Table 15:** *In vitro* drug release data of Nateglinide sustained release tablets (N1 to N13).

Time (hrs)	N1	N2	N3	N4	N5	N6	N7	N8	N9	N10	N11	N12	N13
0	0	0	0	0	0	0	0	0	**0**	0	0	0	0
1	18 ± 3.99	14 ± 3.45	16 ± 3.51	29 ± 3.68	19 ± 4.03	16 ± 4.02	15 ± 3.38	10 ± 3.37	**9 ± 3.90**	8 ± 3.25	12 ± 3.64	10 ± 3.31	10 ± 3.96
2	40 ± 3.46	38 ± 4.14	28 ± 4.07	38 ± 3.72	39 ± 3.72	32 ± 3.61	20 ± 3.64	18 ± 3.99	**15 ± 3.68**	13 ± 3.63	20 ± 3.36	18 ± 3.61	17 ± 3.64
3	56 ± 3.14	59 ± 3.78	49 ± 3.54	54 ± 3.58	48 ± 3.80	41 ± 3.94	31 ± 437	26 ± 3.42	**26 ± 3.42**	24 ± 4.01	31 ± 4.24	29 ± 3.64	30 ± 3.76
4	78 ± 4.44	72 ± 3.45	61 ± 3.92	79 ± 4.45	61 ± 4.19	59 ± 4.55	42 ± 4.11	37 ± 3.98	**32 ± 4.20**	30 ± 4.25	39 ± 3.81	37 ± 4.29	38 ± 3.22
5	85 ± 3.83	83 ± 4.42	75 ± 3.96	94 ± 3.83	80 ± 3.27	73 ± 3.93	53 ± 3.67	48 ± 3.71	**39 ± 3.63**	37 ± 3.45	47 ± 3.53	45 ± 4.23	47 ± 3.61
6	97 ± 4.56	90 ± 4.28	84 ± 3.52		95 ± 3.36	85 ± 4.19	61 ± 4.10	59 ± 4.45	**47 ± 3.06**	45 ± 3.23	54 ± 3.69	53 ± 3.98	54 ± 3.83
7		95 ± 4.01	90 ± 4.08			91 ± 3.40	69 ± 3.36	65 ± 3.23	**53 ± 3.67**	51 ± 3.64	62 ± 3.58	59 ± 3.87	60 ± 4.09
8			96 ± 3.81			98 ± 3.79	78 ± 3.44	78 ± 3.44	**60 ± 3.27**	58 ± 4.21	79 ± 4.34	70 ± 4.01	71 ± 3.56
9							97 ± 3.43	85 ± 3.87	**75 ± 4.14**	65 ± 3.82	88 ± 3.46	81 ± 3.97	82 ± 4.13
10								98 ± 3.30	**81 ± 3.74**	74 ± 4.30	98 ± 3.97	90 ± 3.64	96 ± 3.98
11									**92 ± 3.48**	80 ± 4.16		99 ± 4.15	
12									**96 ± 3.92**	89 ± 4.43			

**Table 16:** Evaluation parameters of bilayer tablet.

Name	Weight variation (%)	Hardness (kg/cm^2^)	Thickness (mm)	Friability (%)
Bilayer tablet	0.54	6.5	5.36	0.48

**Table 17:** *In vitro* drug release data of Nebivolol and Nateglinide bilayer tablet.

	Nebivolol (NBL8)	Nateglinide (N9)
Time (min)	% drug release	
0.1 N HCl		
0	0	0
5	25 ± 3.45	1.8 ± 1.01
10	48 ± 3.37	2.4 ± 1.23
15	69 ± 4.28	3.1 ± 1.21
20	84 ± 4.61	3.6 ± 1.34
25	91 ± 4.02	4.1 ± 1.28
30	97 ± 3.43	4.9 ± 1.46

Time (hrs)	% drug release	
0.01 N HCl with 0.5% w/v SLS		
1		8 ± 3.46
2		17 ± 3.69
3	—	25 ± 4.29
4	—	36 ± 3.66
5	—	45 ± 3.46
6	—	53 ± 4.32
7	—	64 ± 4.23
8	—	72 ± 3.70
9		80 ± 3.42
10	—	87 ± 4.18
11		91 ± 3.42
12	—	97 ± 3.22

**Table 18:** Kinetic data analysis of Nebivolol (immediate release) layer in bilayer tablet.

Zero order		First order		Higuchi		Korsemeyer	
Time (min)	Cum. %release	Time (min)	Log cum. %remaining	Sqrt. of time	Cum. %drug release	Log of time	Log cum. %release
0	0	0	0	0	0	0	0
5	25.45	5	1.872447648	2.236067977	25.45	0.698970004	1.405687787
10	48.37	10	1.712902125	3.16227766	48.37	1	1.684576087
15	69.28	15	1.487421211	3.872983346	69.28	1.176091259	1.840607879
20	84.61	20	1.18723862	4.472135955	84.61	1.301029996	1.927421695
25	91.02	25	0.953276337	5	91.02	1.397940009	1.959136831
30	97.43	30	0.409933123	5.477225575	97.43	1.477121255	1.988692703

**Table 19:** Kinetic data analysis of Nateglinide (sustain release) layer in bilayer tablet.

Zero order		First order		Higuchi		Korsemeyer	
Time (hr)	Cum. %release	Time (hr)	Log cum. %remaining	Sqrt. of time	Cum. %drug release	Log of time	Log cum. %release
0	0	0	0	0	0	0	0
1	9.9	1	0	1	8.46	0	0.927370363
2	15.68	2	1.96161091	1.4142136	17.69	0.301029996	1.247727833
3	26.42	3	1.9154526	1.7320508	25.29	0.477121255	1.402948829
4	32.2	4	1.87337874	2	36.66	0.602059991	1.564192461
5	39.63	5	1.80167806	2.236068	45.46	0.698970004	1.657629431
6	47.06	6	1.73671513	2.4494897	53.32	0.77815125	1.726890141
7	53.67	7	1.66913085	2.6457513	64.23	0.84509804	1.807737922
8	60.27	8	1.55351894	2.8284271	72.7	0.903089987	1.861534411
9	75.14	9	1.43616265	3	80.42	0.954242509	1.905364069
10	81.74	10	1.29181269	3.1622777	87.18	1	1.940416865
11	92.48	11	1.10788803	3.3166248	91.42	1.041392685	1.961041217
12	96.92	12	0.93348729	3.4641016	97.22	1.079181246	1.987755617

**Table 20:** Stability studies.

S. number	Parameters	Conditions			
		Initial	40°C/75%RH		
		0 days	1 month	2 months	3 months
		Nebivolol and Nateglinide bilayer tablet			
1	Average weight (mg)	450 ± 5.0	450 ± 5.0	450 ± 5.0	450 ± 5.0
2	Hardness (kg/cm^2^)	6.5 ± 0.5	6.5 ± 0.5	6.3 ± 0.5	6.0 ± 0.5
3	Thickness (mm)	5.36 ± 0.04	5.34 ± 0.05	5.34 ± 0.08	5.34 ± 0.08
4	Dissolution (cum.% drug release)				
	Nebivolol (30 min)	97 ± 3.43	97 ± 3.15	96 ± 3.24	96 ± 3.65
	Nateglinide (12 hrs)	97 ± 3.22	97 ± 3.83	96 ± 3.67	96 ± 3.48
